# Primary Healthcare Centre Improvement and Privatisation in the Kingdom of Saudi Arabia: A Systematic Review

**DOI:** 10.7759/cureus.76357

**Published:** 2024-12-25

**Authors:** Zainab Albaher, Heba Alqurashi

**Affiliations:** 1 Quality Management, Tadawi General Hospital, Dammam, SAU; 2 Public Health, Saudi Electronic University, Dammam, SAU

**Keywords:** management, policies, primary healthcare centers, saudi arabia, systematic review

## Abstract

Saudi Arabia prioritises primary healthcare reform to address challenges like population growth, high demand, high costs, and unequal access. The 2030 vision aims to integrate and maintain primary healthcare centre (PHC) services, while the healthcare privatisation plan seeks to modernise and expand primary care, medical cities, and dialysis centres. A search was run on different databases, and 18 studies were included in the review based on inclusion and exclusion criteria. Data were extracted from the studies, which included the aim of the study, sample characteristics, study characteristics, and outcomes. This systematic review integrated findings from 18 recent studies on Saudi PHC services, infrastructure, workforce, policies, and reforms. The evidence depicts a system with strengths in some areas like immunisation but substantial gaps in critical domains like chronic disease management, preventive care, health information systems, care coordination, and equity. Patients face organisational, geographic, and socioeconomic barriers that impede PHC access and force overreliance on emergency services. Moreover, the PHC system faces significant gaps in areas like preventive care, health information systems, care coordination, and equity, with patients facing organisational, geographic, and socioeconomic barriers that impede access and overreliance on emergency services. Workforce limitations, particularly in rural areas, contribute to suboptimal quality. Strengthening PHCs is crucial for achieving Saudi Vision 2030 goals of improved population health outcomes, user experience, care integration, and healthcare sustainability.

## Introduction and background

Primary healthcare centres (PHCs) are considered the patient's first point of contact with the healthcare system. Several nations use health indicators (service availability, drug availability, availability of health workers, access to radiology machines) to assess the quality or performance of their primary healthcare system [1]. The PHC program in Saudi Arabia was launched in 1983, based on the WHO concept of "Health for All" as stated in the "Alma Ata" proclamation. The Saudi government has prioritised PHC over time. According to the Saudi MOH, there were 2,325 PHC centres scattered throughout the Kingdom in 2016. Approximately 7% (162) of these facilities are in the Al-Madinah region [2].

Governments that have prioritised primary healthcare have achieved significant progress toward the Sustainable Development Goals (SDGs) agenda, whether they are resource-rich or resource-poor [3]. Saudi citizens and foreigners employed in the public sector in Saudi Arabia are provided with basic medical services via the primary healthcare sector. Without first considering the primary healthcare services at the system's core, no reform of the Saudi Arabian healthcare system can be fully accomplished [3].

Saudi Arabia’s health sector, like those of many other nations worldwide, is facing serious difficulties as a result of the country's expanding population, higher demand for healthcare services, expensive healthcare costs, unequal access, and worries about the quality and safety of care [1]. As a result, the sector is rapidly changing in Saudi Arabia following the nation's National Transformation Program and as part of Saudi Arabia's Vision 2030 for the future [1]. These three pillars are: (1) facilitating access to health services; (2) enhancing the quality and efficiency of health services; and (3) promoting health risk prevention. To achieve these objectives, Saudi Arabia has already begun to shift its attention and financial resources away from secondary and tertiary healthcare institutions and toward the reform and restructuring of primary healthcare [1]. The present illness burden, which is increasingly made up of non-communicable diseases, is still another reason why KSA should prioritise healthcare investment in enhancing primary and preventive care to attain efficiency and value for money. With the new model of care, the proposed changes anticipate this priority, which is why it is critical to determine the national preparedness of PHCs to adopt such reforms [1].

The study critically evaluated the key issues with the Saudi PHC system, which can improve healthcare access and quality and align with governmental development objectives. It will serve as a valuable resource for decision-makers and medical professionals to improve the Saudi healthcare system. Additionally, the study analyses best practices in primary healthcare and identifies potential strategies that can be implemented across Saudi Arabia. This will help to ensure that the country's healthcare system is not only aligned with global standards but also addresses the unique needs and challenges of its population. Moreover, the study highlights the importance of collaboration between different stakeholders, including government agencies, healthcare providers, and community organisations, to create a comprehensive and sustainable primary healthcare system in Saudi Arabia.

## Review

Methods

Research Framework

For conducting a systematic literature review, the researchers followed Kitchenham's (2004) guidelines, which are based on three existing guidelines for SR, i.e., 1) The Cochrane Reviewer’s handbook, 2) Preferred Reporting Items for Systematic Reviews and Meta-Analyses (PRISMA) guidelines, and 3) The Australian National Health and Medical Research guidelines [4]. In the first step, journals and articles from different sources are searched through keywords. 

Search Strategy

The research was carried out between June 2023 and November 2023, as data on the Saudi healthcare system were collected from published literature in the following databases: PubMed, MEDLINE, CINAHL, Saudi Medical Journal, Eastern Mediterranean Health Journal, and the Saudi Ministry of Health portal.

The keywords used include 'primary care,' 'primary health care,' 'privatisation,' 'quality improvement,''saudi,' ‘and 'transformation and vision 2030'. Also, Medical Subject Headings (MeSH) phrases "Primary Health" AND "Saudi" as well as "Primary Care" AND "Saudi" were used.

The literature searches were augmented first by papers that didn't meet the eligibility criteria and were discarded after the titles and abstracts were examined. Based on inclusion-exclusion criteria, full-text articles were filtered out in the second step. Additionally, a senior member of the team was contacted for dispute resolution if there were any issues.

Inclusion and Exclusion Criteria

Both published literature and grey literature having observational, cohort, cross-sectional, case-control, and randomised/non-randomised designs were included if they met the inclusion/including the originality of research conducted between 2015 and 2023; if they had data related to privatisations and primary healthcare, both qualitative and quantitative study designs were included.

Studies were excluded if they were conducted outside Saudi Arabia and related studies using a systematic review design. The irrelevant and relevant studies are included and excluded based on the Joanna Briggs Institute (JBI) assessment. The articles were published in languages other than English.

Risk of Bias

The present review used the JBI tool known as the “JBI Critical. Appraisal Checklist” to assess the studies [5]. Each study was assessed on nine domains. Each domain was assessed as having either yes/no/unclear/not applicable, and a final judgement was made as either include/exclude/seek further information. The final risk of bias assessment for each study was a combination of assessments on each domain as per the instructions given in the tool [5].

Data Extraction

Extracted information included study characteristics (author, year, journal, city/country, aim of the study described in the Population (P), Exposure (E), Comparator (C), Outcome (O), study design, sample characteristics, privatisation, and conclusion of the study).

Results

**Figure 1:**
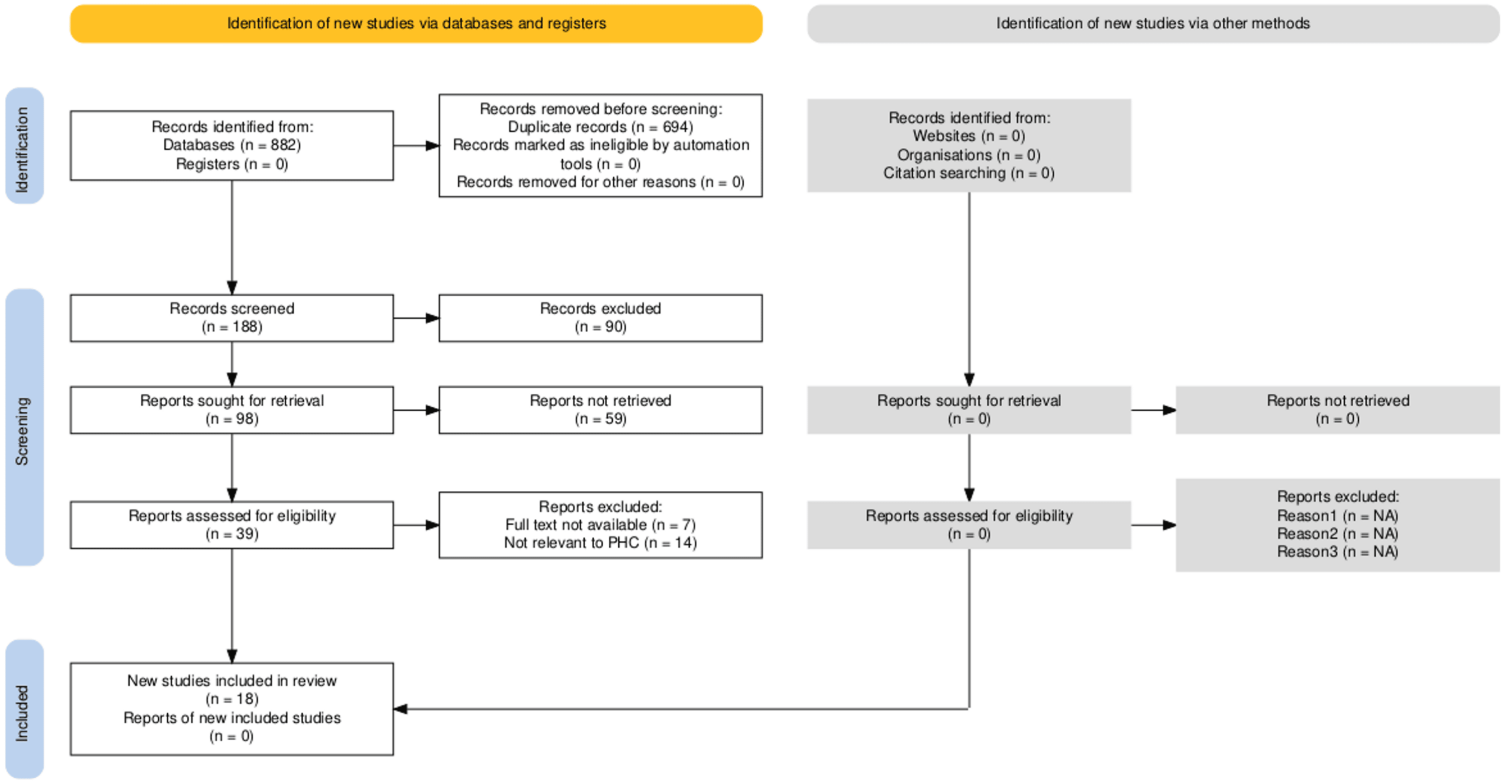
PRISMA flow diagram PRISMA: Preferred Reporting Items for Systematic Reviews and Meta-Analyses; PHC: primary healthcare centre

The outcome of the systematic review is presented in Tables 1, 2 for the evaluation of the privatisation in primary healthcare centres' improvement in the context of Saudi Arabia. The PRISMA and JBI assessments were employed in the screening of the research studies, and a total of 18 most relevant research studies were extracted for the systematic review [6]. The complete description of the screening, extraction, and results is presented in Figure 1, and the PRISMA diagram [7] (Figure 1) is as follows:

**Table 1:** Study characteristics DR: Diabetic retinopathy; DM: diabetes mellitus; PHC: primary healthcare centre; NCD: non-communicable disease

Study Year Reference	Study Design	Sample (n)	Aim	Intervention in PHC by privatisation	Outcomes Measured	Results
Alghamdi et al., 2020 [2]	Cross-sectional survey	481 adults	To assess the awareness of patients for utilisation of PHC and discover the factors that encourage or discourage PHC utilisation	Privatisation was not discussed.	Awareness of the existence of primary care centres, awareness of services provided, and utilisation of primary care centres.	Presented as percentages. 83.4% were aware of a primary care centre in their district. Immunisation was the best-known service (79% aware). 30.7% never utilised primary care services. Awareness of primary care services varied, with good awareness of some services like immunisation but poorer awareness of services like mental health. Utilisation was associated with factors like age, gender, and marital status. Dissatisfaction with services was the main reason for non-utilisation.
Dawoud et al., 2016 [8]	Cross-sectional study	300	To determine the extent to which individuals visit the emergency room for non-urgent cases, identify the factors for ER utilisation in cases of non-urgent cases, and understand patients' knowledge about PHC as an alternative to ER.	The role of privatisation was not discussed.	Non-urgent ER usage factors. Patient knowledge about primary care facilities. Reasons to avoid primary care and seek ER overload consequences	53% of ER visits were non-urgent. Singlehood, youth, and poverty boost non-urgent ER visits. Primary care clinics were understood by 90% of patients. Poor services, resources, and availability drove individuals away from primary care facilities. Congestion slowed ER visits by three hours. High non-urgent ER usage needs primary care access and overuse prevention. High non-urgent ER utilisation signals poor primary care. Instruction in ER use may lessen crowding.
Seitan and Gillespie, 2020 [9]	Cross-sectional study	157	To examine the relationship between demographic variables of patients, physician communication quality, and care coordination in PHC	The study mentioned ongoing healthcare reforms in Saudi Arabia to increase privatisation and integration of services.	Patient demographics, physician-patient communication measures, care coordination measures, and overall patient satisfaction ratings.	Demographics don't affect satisfaction. Overall satisfaction was connected to communication indicators (p<0.005) and care coordination indicators (p<0.05), with 81% explained by communication indicators in regression analysis. Patient satisfaction was substantially predicted by physician-patient communication (p<0.005, 81% variation). Saudi PHCs can enhance patient experience and quality by enhancing physician-patient communication.
Aldhamadi et al., 2019 [10]	Cross-sectional survey	382.	To explore the community participation and knowledge and attitude of patients towards PHC and discover reasons behind not utilizing PHC	Privatisation was not discussed.	Public opinions and attitudes towards primary healthcare centres, services, and providers. Reasons not to use primary care initially. Preferred provider for various health issues.	54% preferred primary care for medical issues. 72.3% prioritised emergency treatment over primary care. Unhappy with primary care availability, quality, infrastructure, etc. Saudi public awareness and use of basic healthcare centres were poor. Problems with primary care. Awareness campaigns and quality improvements are needed.
Alomar et al., 2021 [11]	Cross-sectional study	403	To assess the satisfaction of patients towards PHC and identify potential barriers	Privatisation was not discussed.	Patient satisfaction with primary care, barriers to utilising primary care (organisational, socioeconomic, access, patient-doctor relationship).	Satisfaction was 28.3%. Most reported barriers were organisational (54.8%) and socioeconomic (47.7%). Presented as percentages. Lower satisfaction was associated with female gender, younger age, higher education level, and high organisational and patient-doctor barriers. Patient satisfaction with primary care was low in the sample. Organisational and socioeconomic factors were major barriers to primary care utilisation, highlighting areas for improvement.
Jahan et al., 2017 [12]	Descriptive study	655 PHC research articles published in Saudi Arabia from 1983-2011		Not discussed	Number and types of PHC research publications over time, research topics, geographical distribution, and publishing institutions	655 PHC research articles published from 1983-2011. A steady increase in the number of publications over time. 85.6% original research, 93.4% cross-sectional study design. Main topics: chronic diseases (36.4%) and health services research (23.9%) 56.2% published by universities, 24.9% by Ministry of Health and 46.3% from Riyadh province. The number of PHC research publications in Saudi Arabia increased steadily from 1983 to 2011 The majority were original research (85.6%) with cross-sectional study design (93.4%). The main topics were chronic diseases (36.4%) and health services research (23.9%). Most were published by universities (56.2%) and from Riyadh province (46.3%). PHC research output is low but increasing over time in Saudi Arabia. The majority of cross-sectional studies from universities on chronic diseases and health services need capacity building in PHC research and establishing central regulatory authority
Alrasheedi et al., 2019 [13]	Cross-sectional study	620	To examine the association between wait time and patient satisfaction in PHC	Not discussed	Wait times for registration, payment, seeing physician, performing assays, and dispensing medications. Patient satisfaction	27.9% waited 21–30 minutes for the doctor. Patients unhappy with registration/payment wait times: 32.79% and 40.45% of patients dislike medicine distribution wait times. 39.16% were unhappy with vital signs wait times. 37.22% are unhappy with dental consultation wait times 44.34 percent of patients dislike radiology wait times. Patient satisfaction is positively correlated with education, marital status, and employment (p<0.001). Regression was seen between patient satisfaction and age (p = 0.002) and literacy (p<0.001). Patient satisfaction decreases with longer wait times. Patients are unhappy with medicine, vital signs, dentistry, and radiological wait times. Education, marital status, and employment increase patient satisfaction.
AlMubarak et al., 2021 [14]	Qualitative case study	21		Privatisation aims to reduce public health expenditure, improve access and quality of care, and increase efficiency, which resulted in the establishment of autonomous health clusters.	Perceptions of privatisation	Privatisation granted health clusters more autonomy from the government. Privatisation introduced accountability for hospitals to reduce waste. Privatisation encouraged cooperation between hospitals in a cluster but competition with private hospitals. Participants perceived conflict between concepts of autonomy and accountability. Unclear implementation of privatisation at the hospital level.
AlHargan et al., 2019 [15]	Cross-sectional study	280	Awareness of patients towards DR, practice of regular eye checkups, DM control among diabetic patients	Not discussed	Awareness of diabetic retinopathy. Practice of regular eye exams. Diabetes control	88% know diabetes affects the retina 76% know blood sugar management lowers diabetic retinopathy risk. 66% know diabetic retinopathy may blind. 45% got eye exams last year. Individuals with high school education are more aware than those without formal education (p<0.001). Higher awareness in income >10,000 vs ≤5,000 SAR (p<0.05). Improved medication adherence and home blood sugar monitoring in aware patients (p<0.001). Despite high diabetic retinopathy awareness, just half received yearly eye examinations. Awareness increases with education and affluence. Awareness improves diabetes medication adherence and monitoring.
Bawazir et al., 2019 [16]	Cross-sectional survey	41	Access the readiness and capacity of PHC for preventive measures and implementation of non-communicable diseases	Not discussed	Availability of human resources, medical equipment, infrastructure, medicines, service utilisation, referral systems, and community outreach	About 90% of PHCs were urban. Low NCD prevention and control staff training, especially for nurses and other providers PHCs provide most diagnostics, testing, prescriptions, and referrals. PHCs may include NCD prevention and management in everyday operations. Staff training on NCDs and clear rules and instructions for NCD prevention needs improvement.
Almugti et al., 2023 [17]	Cross-sectional study	377	To assess the knowledge skills and practices of PHC workers, determine the satisfaction of PHC workers in the surveillance system	Not discussed	Knowledge, skills, and practice of primary healthcare workers regarding disease surveillance system; satisfaction with surveillance system	88% reported no infectious infections last year. 48% were unsure which diseases to report immediately. 57% had trouble diagnosing leishmaniasis. 44 percent considered notification forms complex and time-consuming. Most primary care personnel did not report infectious diseases. Identification and reporting of priority illnesses were lacking in knowledge and expertise. Frustration with cumbersome forms and poor notice feedback
Almujadidi et al., 2022 [18]	Qualitative study using interviews and focus group	17	To identify barriers and enablers for addressing social determinants of health in the clinical setting of Saudi Arabia	Not discussed	Barriers and enablers for addressing social determinants of health (SDH) in primary care settings	Financial problems, family relationships, mental health, and ageing issues are common in Saudi primary care. Lack of medical expertise, organisational restrictions, cultural norms, and unclear physician roles hinder SDH treatment. SDH training, identification streamlining, interprofessional collaboration, and community advocacy enablers.
Al-Sheddi et al., 2023 [19]	Cross-sectional study	20 health regions	To access the equity of PHC distribution	Not discussed	Number of primary healthcare centres, population, ratio of PHCs per 10,000 population, Gini index, and Pearson correlation coefficient	The ratio of PHCs per 10,000 people declined from 0.72 in 2017 to 0.62 in 2021. A Gini index of 0.27 indicated relative equality. Decrease in PHCs per 10,000 population over time; Gini index demonstrated relative equality; positive association between population and PHCs in 3 regions, negative correlation in 13 regions. PHCs per 10,000 people fell from 2017 to 2021. In 20 health areas, the Gini index demonstrated a generally equitable distribution of PHCs. Provinces have different PHC to population ratios. PHC distribution must be equal for healthcare access.
Hazazi et al., 2022 [20]	Cross-sectional survey	315	To examine the experience and satisfaction of patients with non-communicable diseases who received care from PHC	Not discussed	Patient experiences, satisfaction with care, and preferences for chronic disease management location	71.4% of patients had no problem getting diagnostic testing, 72.7% treatment, and 67.6% specialists. 76.8% were prescription-free. 41.6 percent received no medication list, and 54.1% received no written self-management instructions. Seniors are more likely to get medication lists (p<0.01). 75.5% discussed work/family/illness; 91.1% said providers communicate effectively. Only 68.2% of 91.7% rated values/traditions were addressed. Organising care satisfaction: 78.7%. 31.7 percent never addressed treatment goals, and 37. percent never mentioned care goals. 52.1 percent not encouraged to attend support groups, and 39.6% are not socially supported 30.1 percent lack self-care instructions, and 47.5% lack monitoring logs.
Haji, 2023 [21]	Cross-sectional survey	306 family medicine residents in Saudi Arabia	To assess the attitude of family medicine residents toward TBC and its associated factors	Not discussed	Attitudes of family medicine residents toward team-based care	The average attitude score was 2.71/5. The average attitude score was 2.71/5. Most points are for team value (3.94), least for efficiency (2.47), and physician's shared role (1.71). Team value scores were considerably higher among TBC practitioners. Non-significant differences by gender, residence, cluster, and network participation. Residents were generally enthusiastic, especially about teamwork. Training and role models are needed to promote physician teamwork and understanding.
Al-Rebdi et al., 2021 [22]	Cross-sectional study	358	To assess the awareness of PBC among the visitors and the source of that awareness	Not discussed	Awareness of patient rights and patient bill of rights	72% showed moderate patient rights awareness, and 65% were uninformed of the patient bill of rights. Education, wealth, and hospital follow-ups greatly affect awareness. Medical professionals are the major rights sources. Respect and care are well-known, but autonomy, consent, and secrecy are not. Despite considerable awareness, the Saudi Patient Bill of Rights is unknown to most. Raise awareness of patient rights and the Bill of Rights.
Alnasser et al., 2023 [23]	Retrospective analysis of electronic medical records		To determine the extent and reasons for nonurgent cases to visit the ED, predictors, and patient outcomes	Not discussed	Non-urgent emergency department visit characteristics	61.4% of ED visits were non-urgent (CTAS 4-5). Routine exams/investigations (40.9%), medicine refills (14.6%), upper respiratory infections (9.9%). 73.4% weekdays, 94.2% medicines, 62.8% lab tests, and 4.7% sick leave. Not many are needed for admission, consultation, or referral. A high percentage of non-urgent ED visits suggests primary care management needs improvement. Education, restrictions, and better primary care access are needed to minimise non-urgent ED utilisation.
Alfaqeeh et al., 2017 [24]	Questionnaire survey	935	To determine the factors influencing the access and utilisation of PHC in urban rural areas of Riyadh.	Not discussed	Barriers and enablers to accessing primary healthcare services	Rural patients were wealthier, had more tests, and waited longer. Many urban and rural patients are satisfied with PHCs. Rural patients were wealthier, had more tests, and waited longer. Rural patients battled with distance; urban patients wanted more hours. Low rural PHC cleanliness. Patients in rural areas were better protected. Saudi urban and rural patients utilise PHC differently. Distance, cleanliness, and prevention may reduce rural-urban gaps.

**Table 2:** Quality assessment by the Newcastle-Ottawa scale of cross-sectional studies

Study Year Reference	Representativeness of Sample	Sample Size	Non-respondents	Ascertainment of Exposure	Comparability	Ascertainment of Outcome	Statistical Test	Total Score (Out of 10)	Quality Rating
Alghamdi et al., 2020 [2]	1	1	0	2	2	2	1	9	High
Dawoud et al., 2016 [8]	1	1	0	1	2	1	1	7	Moderate
Seitan and Gillespie, 2020 [9]	1	1	0	1	2	2	1	8	High
Aldhamadi et al., 2019 [10]	1	1	0	1	1	1	1	6	Moderate
Alomar et al., 2021 [11]	1	1	1	1	1	2	1	6	Moderate
Alrasheedi et al., 2019 [13]	1	1	0	1	1	1	1	6	Moderate
AlHargan et al., 2019 [15]	1	0	0	1	1	1	1	5	Moderate
Bawazir et al., 2019 [16]	1	1	0	1	0	2	0	5	Moderate
Almugti et al., 2023 [17]	0	1	1	0	2	1	0	4	Low
Al-Sheddi et al., 2023 [19]	1	1	0	1	0	2	1	6	Moderate
Hazazi et al., 2022 [20]	1	1	1	0	2	2	1	8	High
Haji, 2023 [21]	0	1	1	1	0	1	0	4	Low
Al-Rebdi et al., 2021 [22]	2	1	1	1	0	1	1	7	Moderate

PHC Infrastructure and Services

Al Saffer's article (2021) examined PHCs in Saudi Arabia in detail, including the fundamental components required for efficient provision of health care [1]. It revealed distinctions among urban and rural locations in the geographical distribution of PHCs, the supply of resources, and the services provided, underscoring the need for customised strategies. The research highlights the need for PHC certification, round-the-clock functioning in rural regions, and improvements in e-health to improve the efficacy of the healthcare sector. It also sheds light on the paucity of specific medical specialisations and the widespread use of paper-based recordkeeping [1].

PHC Facility Distribution and Density

Equitable access to PHCs requires proper geographic distribution of facilities relative to population needs. Al-Sheddi et al. (2023) analysed the number of PHCs across all 20 health regions in Saudi Arabia from 2017 to 2021 [19]. The overall ratio of PHCs decreased slightly. Rural centres had lower density but more examination rooms than urban centres. The Gini index of 0.27 indicated a relatively equal distribution of PHCs nationally. While the overall PHC density was 0.63 centres per 10,000 populations, substantial variability existed across regions from 0.32 to 1.56 per 10,000. This demonstrates geographical disparities in PHC infrastructure distribution, especially affecting remote areas.

Medical Equipment and Resources

The widespread accessibility of medical equipment influences the ability to serve in addition to infrastructure. PHC facilities must be outfitted with uniform diagnostics and therapeutic technologies across the country, which requires strategic expenditures. Al Saffer's study (2021) highlighted the importance of licensing regulations in influencing access to vital medical devices such as radiological machinery, as well as in enhancing the reach of amenities and health professionals. It illustrates how basic healthcare institutions' certification enhances their preparedness and capability and emphasises the significance of policies in guaranteeing the supply of vital medical supplies [1].

Health Information Systems

Systems for tracking and enhancing personal health data are essential for PHC treatments. Jahan and Al-Saigul's (2017) investigation of the main healthcare environment in Saudi Arabia shed light on the integration of Health Information Systems (HIS) as a crucial factor that is frequently crucial to the efficiency of the health system [12]. The results suggest that there is room for improvement in technology within the Saudi healthcare system. Specifically, the implementation of thorough HIS could have a substantial positive impact on the treatment of patients, distribution of resources, and development of policies in PHCs.

Accreditation

By the year 2021, the Central Board for Accreditation of Healthcare Institutions (CBAHI) had accredited 300 PHC facilities in Saudi Arabia. The Al Saffer (2021) study's key result demonstrates that the study recognised how credentialing affects PHCs. It was emphasised that the CBAHI's licensing of these facilities has a positive impact on the accessibility of medical personnel, offerings, and equipment such as radiography scanners. This emphasises how important accreditation standards are to improving Saudi Arabia's fundamental medical care institutions' capability and preparedness [1].

Seitan and Gillespie's study (2020) reported that the general level of fulfilment was shown to be highly affected by the calibre of care integration and physician-patient engagement [9]. The study made clear that strengthening physician-patient engagement is a critical component for enhancing patient satisfaction and treatment standards at PHCs. To enhance the efficacy, safety, and standard of treatment, it also suggested actions, including guaranteeing patient translation services and adding higher communication guidelines to the certification requirements established by the Saudi CBAHI.

PHC Reforms Under Vision 2030

Haji (2023) noted that the comprehensive medical care changes, carried out under the umbrella of Saudi Vision 2030, have their eyes set on a significant overhaul of PHCs [21]. Acknowledging the shortcomings of existing PHCs, attention is being focused on a thorough redesign. The suggested Model of Care, outlined in Vision 2030, places a strong emphasis on forward-thinking, patient-centred medical treatment. However, current issues, including an uneven standard of care, ineffective communication, and patient discontent, led to a tactical change. The team-based treatment strategy was established by the Assistant Deputy for Primary Healthcare at the Saudi Ministry of Health to overcome these obstacles.

Al Saffer et al. (2021) noted that the Kingdom of Saudi Arabia is aware of how important primary healthcare is to the improvement of the nation’s medical provision [1]. The country has strategically started transformations aimed at three main areas within the guidelines of Vision 2030: increasing access to medical services, boosting wellness preventative measures, and strengthening operational quality and effectiveness. This change affects a wide variety of services offered by PHCs, including preventative care, medicines, illness administration, and wellness promotion.

PHC Service Delivery and Quality Service Availability and Standards

Numerous studies have assessed the PHC establishments' service quality and accessibility. Alfaqeeh et al. (2017) highlighted the significant disparities between these places in terms of factors like poverty, schooling, health care, and preventative care [24]. Eliminating the incidence of chronic illnesses in Riyadh province using enhanced PHC delivery of services would largely depend on how well these disparities are addressed in the development of strategies and initiatives aimed at closing the disparities in medical services between rural and urban areas [24]. Alnasser et al. (2023) concluded that the PHC framework in Saudi Arabia offers free and limitless medical treatment to its inhabitants, greatly enhancing their accessibility to healthcare [23]. This progress has been supported by initiatives such as round-the-clock centre operation and simple scheduling choices using the MAWID app and 937 contact centres. Although the patient contentment resulting from these activities has increased, Saudis have a propensity to visit.

Haji (2023) suggested that by putting wellness and preventative measures at the forefront of healthcare, they hope to enable people to take ownership of their health using the "Model of Care." However, there are issues with the delivery of care, standardisation, and overall quality in the PHC schemes that are already in place [21]. A team-based care strategy has been put out as a solution to this problem, in line with Vision 2030 objectives. By empowering family doctors to oversee teams using programs like "A Physician for Every Family," this strategy seeks to raise the standards and availability of PHCs.

Investigation by AlMubarak et al. (2020) noted that the Saudi Arabian government's deployment of the Privatisation Program in the healthcare sector, particularly in PHC, is a calculated reaction to the mechanism's current difficulties [14]. The initiative aims to bring the commercial industry into healthcare delivery to increase the quality of service, connectivity, and effectiveness [14]. According to Bawazir et al. (2019), Saudi Arabia has been aggressively tackling the growing issue of NCDs by emphasising raising standards and PHC delivery of services [16]. Through adherence to the WHO Global NCD Action Plan and evaluations carried out at PHCs under the Ministry of National Guard-Health Affairs. These initiatives demonstrate the nation's strategic method for incorporating NCD remedies into PHC amenities, highlighting the nation's dedication to improving care availability and quality in the fight against NCDs [16].

Hazazi and Wilson (2022) observed that high positive feedback rates at PHC establishments in Saudi Arabia highlighted the excellent standard of treatment that doctors and other medical professionals offer [20]. Although levels of contentment are still high, worries regarding healthcare availability are still present. The results nevertheless underscore the necessity of strengthening the medical system's emphasis outside PHCs to provide a more all-encompassing and holistic approach to treatment, stressing the significance of patient empowerment in illness management. This implies that doctors have a crucial role to play in providing guidance and opening doors to PHCs, promoting a more patient-centred and coordinated method of treatment throughout Saudi Arabia's PHC system [20].

Referrals and Care Coordination

Connecting patients across PHC and specialist services is vital for comprehensive care. Almujadidi et al. (2022) argued that by progressively introducing the idea of social determinants of health (SDoH) into PHCs, Saudi Arabia is improving patient connections between PHCs and speciality therapies that provide all-encompassing treatment [18]. There are still obstacles to be addressed, though, such as the sluggish acceptance of such modifications, frequent visits without resolving fundamental social concerns, and the perception among medical personnel that they lack the necessary training or qualifications to address patients' SDoH [18].

Likewise, the Saudi Health Electronic Surveillance Network (HESN) technology has strengthened the capacity of healthcare providers to improve patient connectivity between PHCs and specialised services for all-encompassing treatment. Nonetheless, the primary healthcare personnel face difficulties, including a lack of expertise and experience, as well as discontent with the communication procedures' apparent complexity and response procedure [17]. According to Al-Sheddi et al. (2023), by enhancing coordination and referrals among PHCs and medical specialists, Saudi Arabia has made tremendous progress in enhancing care for patients [19]. The Ehalati system, which was implemented in 2017, has streamlined the process of transferring patient data from general to more specialised care physicians.

Seitan and Gillespie (2020) observed that PHCs play a critical role in facilitating patient treatment across specialities in Saudi Arabia and supporting an all-inclusive system of healthcare [9]. The study's conclusions highlight the need for strong doctor-patient communication as a pillar for improving patients' experiences in PHCs as a whole. Additionally, the study noted areas that needed advancement, particularly in enhancing follow-up protocols and clinician knowledge of patient medical histories [9].

Interpersonal Care and Patient-Centredness

The PHC approach prioritises continuous, person-centred care with a focus on doctor-patient relationships. However, PHC provider interpersonal and communication quality was suboptimal in Saudi Arabia [20]. Another study reported that participants felt reforms promoted accountability to reduce waste but conflicted with patient-centred values by restricting physician time and decision-making autonomy [21]. Balancing efficient resource use with personalised care remains an ongoing challenge.

Health Education and Prevention

Health education and disease prevention are fundamental PHC components. Almugti et al. (2023) reported that 48% of PHC workers had poor knowledge of diseases requiring immediate reporting for prevention [17]. Enhanced health education activities and staff training on prevention are imperative to improve population health outcomes. The assessments reveal variable but overall suboptimal PHC service quality in Saudi Arabia. However, studies demonstrate access barriers within Saudi Arabia’s PHC system. Spatial accessibility and geographic proximity to PHC services are a prerequisite for utilisation. Rural residents face greater spatial barriers, with distance and lack of transportation impeding PHC access compared to urban areas [24].

Temporal Accessibility and Utilisation

Operating hours are another dimension of access. Alfaqeeh et al. (2017) found that urban residents had a higher demand for after-hours services than rural residents [24]. However, AlMubarak et al. (2021) indicated that recent PHC reforms aimed at accountability and efficiency have restricted physician appointment times, which could conversely impact timely access [14]. Monitoring and optimising operating hours based on community needs are important to ensure temporal accessibility.

Regarding utilisation, Dawoud et al. (2015) reported high rates of 53% non-urgent emergency department (ED) visits by PHC-appropriate conditions, signalling substantial unmet primary care needs [8]. The study found patients avoided PHCs due to perceptions of poor resources, availability, and quality. Patients with unmet PHC needs turn to EDs, contributing to crowding. According to Alghamdi et al. (2020), improving the use of PHCs in Al-Madinah, Saudi Arabia, necessitates tackling important variables that affect patient understanding and involvement with services [2]. Even though a sizable fraction of the populace (30.7%) is aware that PHCs exist, very few use them. Connectivity to PHC clinics was one factor that encouraged usage, although treatment disappointment served as a disincentive.

Affordability

Affordability is a critical healthcare access factor, though not studied for Saudi PHCs specifically. According to Alrasheedi et al. (2019), in Saudi Arabia, improving the affordability and usage of PHCs includes not just financial availability but also the effectiveness of service delivery [13]. Patient fulfilment at PHCs was examined in this study, which was carried out in the Al Qassim locality. The main areas of discontent that were found were the wait times for prescription dispensing, evaluations, and consulting. The goal is to decrease such wait times and enhance patient satisfaction holistically by resolving these issues through technological developments, enough personnel, and user-centred methods. This development is in line with increased PHC usage and improves the general public's access to and use of medical services.

Acceptability

AlMubarak et al. (2021) revealed perceived conflicts between efficiency-orientated reforms and patient-centred care principles [14]. Reorienting PHC culture through training, incentives, and systems to prioritise continuous, holistic, patient-centred population health is critical for a skilled PHC workforce. According to Aldhamadi et al. (2019), PHC acceptance in Saudi Arabia is a complicated matter affected by several variables [10]. Even though a sizable section of the public agrees that PHCs provide high-quality treatment, underutilisation is caused by a pervasive, unfavourable image. The main obstacles are a lack of specialised offerings and a shortage of confidence in medical professionals. Raising the public's understanding through initiatives that highlight the advantages and all-inclusive nature of PHC services may cause perceptions to change, encouraging a greater tolerance and use of these services, which would subsequently lessen the demand on emergency personnel and improve the effectiveness of healthcare altogether.

Patient Satisfaction and Experience of Care

Patient satisfaction is widely used to evaluate healthcare quality. Indeed, studies reveal discrepancies between high satisfaction and actual care provision. Alrasheedi et al. (2019) linked longer waiting times to lower satisfaction [13]. Almugti et al. (2023) found poor provider compliance with disease notification protocols despite 90% satisfaction [17]. Hazazi and Wilson (2019) reported high satisfaction alongside suboptimal chronic disease education and support. Satisfaction measures should be supplemented with detailed care experience assessments [20].

Regarding experiences, Al-Rebdi et al. (2021) found that 65% of patients were unaware of patient rights and the Saudi Patient Bill of Rights [22]. Hazazi and Wilson (2019) highlighted gaps in chronic disease self-management support [20]. Alrasheedi et al. (2019) identified the longest waits for ancillary services like laboratory tests and prescriptions [13]. Concerning experiences persist despite reasonable satisfaction. Satisfaction determinants warrant consideration. Alomar et al. (2021) linked lower PHC satisfaction to the female gender, younger age, higher education, and more perceived barriers [11]. Alrasheedi et al. (2019) associated satisfaction with higher education, employment, and marital status [13]. In summary, while patients report fairly high PHC satisfaction in Saudi Arabia, conflicting evidence on care experiences suggests the need for comprehensive surveys alongside satisfaction measures to identify areas for quality improvement. Capturing diverse patient voices is essential.

Chronic Disease Prevention and Management

Saudi Arabia faces a growing chronic disease burden with PHCs as the frontline for screening, prevention, and management. AlHargan et al. (2019) found that only 45% of diabetic patients received retinal exams in the past year at PHCs in Riyadh, despite risks of blindness [15]. In this regard, Saudi Arabia has made notable progress in improving the management of chronic diseases through PHCs, with a focus on diabetes mellitus (DM) and its related consequences, such as diabetic retinopathy (DR). One important area where the country’s PHC chronic disease administration methods need to be improved is the incentive for diabetes patients to get frequent eye exams.

The PHC system faces a rising chronic disease burden in Saudi Arabia. Hazazi and Wilson (2019) also identified poor self-management support for patients with NCDs [20]. AlHargan et al. (2019) noted suboptimal diabetic retinopathy awareness and screening. PHC capacity for chronic diseases requires strengthening [15]. In summary, studies reveal a heterogeneous picture of Saudi PHC quality, with some strengths but many gaps related to workforce, infrastructure, management, care integration, and chronic disease support.

Access to Care

Ten studies examined PHC access in Saudi Arabia. Dawoud et al. (2016) found high non-urgent ER utilisation, suggesting unmet PHC needs [8]. Patients reported insufficient PHC resources and availability. Rural patients faced greater access barriers like distance and transportation [24]. AlOmar et al. (2021) identified organisational and socioeconomic obstacles to PHC use [11]. Healthcare planners and policymakers must work together to lessen the impact of these barriers by developing solutions that address them. This may entail severely enforcing standards such as proper triage implementation in EDs as well as advertising free services provided at these centres.

Rural-Urban Representation

Rural populations face pronounced PHC access barriers and service gaps [24]. However, no studies focused exclusively on rural PHCs. This reveals a significant knowledge gap regarding rural PHC needs and experiences that must be addressed through dedicated research. PHC provider characteristics were assessed in five studies on healthcare personnel. Haji (2023) and Almugti et al. (2023) reported provider demographics. Haji (2023) surveyed 306 family medicine residents in training, who will staff future PHCs. Almugti et al. (2023) included 377 PHC workers, of whom 45% were nurses, 25% physicians, and 29% administrators. This aligns with nursing predominance in PHC globally (Alliance for Health Policy and Systems Research, 2021).

Ultimately, insights from diverse PHC provider cadres are required to fully strengthen the healthcare workforce. Their realities on the frontlines delivering primary care daily can highlight training gaps plus needed practice improvements.

In summary, sample sizes and locations varied substantially across the 18 Saudi PHC studies reviewed. However, the reporting of detailed demographic data was limited. Only one study explicitly captured both rural and urban communities. No studies focused specifically on populations like women, youth, elderly, or migrant workers with unique PHC needs. This reveals significant knowledge gaps and research opportunities to investigate PHC access, quality, and outcomes across different demographic groups through targeted sampling, disaggregated data reporting, and analysis. Rigorously capturing diverse realities is vital to guide the development of an equitable, patient-centred PHC system meeting the needs of all populations in Saudi Arabia.

Discussion

This narrative synthesis integrated findings from 18 recent studies on Saudi PHC services, infrastructure, workforce, policies, and reforms. The evidence depicts a system with strengths in some areas like immunisation but substantial gaps in critical domains like chronic disease management, preventive care, health information systems, care coordination, and equity. Patients face organisational, geographic, and socioeconomic barriers that impede PHC access and force overreliance on emergency services. Workforce limitations pose a major challenge.

These shortcomings point to the vital need for continued, evidence-based PHC investments and reforms, guided by strong monitoring and evaluation. By 2030, there will be 39.5 million people living in the Kingdom, including 4.63 million people who are 60 or older [25]. By 2030, there will be more people living in urban areas, up from 83.3% in 2016. In comparison to regional and worldwide standards, the Kingdom continues to have a high incidence of preventable injuries and NCDs [26]. To decrease preventable illness and mortality, we must enhance NCD prevention and accident prevention. Significant infectious disease epidemics are still a possibility, particularly during Hajj or in the days following a catastrophe, whether caused by nature or man. 

In the Kingdom, primary healthcare is still poor and uneven. Although there are secondary, tertiary, or specialized hospitals as well as related services all around the nation, there are not enough services for long-term, home, and rehabilitation care [27]. Long-term care (LTC), rehabilitation, and home care are primary areas for diversification and improvement of the healthcare system in the Kingdom of Saudi Arabia (KSA). A primary factor is the evolving demographic profile characterized by a declining fertility rate and an augmented life expectancy. The population aged over 60 is projected to rise from 5.5 percent in 2020 to 11.0 percent by 2030 [28]. By 2030, an extra 20,000 to 22,000 LTC and rehabilitative beds would be necessary. To meet OECD average standards, the country will need an additional 28,000 to 30,000 beds by 2030 [29]. The level of patient care is inconsistent in important ways. The majority of this is brought on by the absence of standardised treatment plans and pathways as well as the inadequate oversight of patient processes and results. The Saudi CBAHI discovered several shortcomings in hospitals of all types in 2015 during the Essential Safety Requirements Survey [27].

The private sector in Saudi Arabia has been engaged in a range of activities related to the direct provision of healthcare services, administration, and management of healthcare facilities, manufacturing of healthcare products, and financing of the healthcare systems [30]. They are carried out by a variety of non-state actors, such as national and international corporations, non-governmental organisations, non-profit organisations, and private people who serve as general practitioners and consultants in the delivery of healthcare services [31,32]. To finance and supply healthcare goods and services, there appears to be an increasing reliance on private actors [33]. This shift towards the private sector in healthcare financing and delivery has raised concerns about equity, as it may result in unequal access to quality care for those who cannot afford private services [34,35]. A study by Gupta et al. found that private equity was associated with lower quality scores regarding patient antipsychotic medication use, patient mobility metrics, and inadequacies. In the same study, they also reported that private equity is significantly correlated with increased mortality (P < 0.05) [36]. The private sector predominates in the healthcare system, leaving the impoverished to contend with limited access to fee-for-service care, which is typically of low quality [32].

Additionally, the involvement of non-state actors in healthcare systems can introduce market dynamics that prioritise profitability over patient welfare, potentially compromising the overall quality and affordability of care. Due to the public sector's management inefficiency, consumers' dissatisfaction with public sector services [37], the private sector's organisational behaviour [32], improved access and equity performance [33], a better supply of drugs and responsiveness [32], higher patient satisfaction or competence [38], and the potential for innovation and technological advancements, the private sector can play a crucial role in improving the overall quality and affordability of healthcare. However, it is important to carefully regulate and monitor the private sector to ensure that it does not prioritise profit over patient care, as this could lead to increased costs and compromised quality of care [34]. It is also evident that persons utilising privately offered healthcare generally possess greater resources and better health [39,40]. As a result, it will be challenging to account for the bias of healthier patients being chosen in private hospitals and achieving better results due to their underlying health status rather than the quality of care. The effect of ownership is only one aspect of the rationale for privatisation [41]. Likewise, private equity ownership can provide significant managerial skills, diminish operational inefficiencies, capitalise on economies of scale, and enhance healthcare access by aligning business motives with the delivery of high-quality treatment [35]. 

Vision 2030 and the Healthcare Transformation Strategy

The Ministry of Health is progressively creating autonomous healthcare groups in various areas, each customised to the capabilities and demography of the local community. These clusters operate as cohesive networks under the direction of advisory groups that handle clinical oversight and decision-making processes [31]. The government intends to privatise certain healthcare facilities, including hospitals and primary health centres, while maintaining ownership and oversight. Subsequently, these facilities will be administered by the private sector. The reform of providers encompasses, through an initial "cluster" phase, the development of all existing Ministry of Health providers into approximately 20 vertically integrated, geographically defined "Accountable Care Organisations (ACOs)." The ACOs will be established as "corporatised" public bodies with substantial and explicitly defined "decision rights." Boards will be established in each ACO to assume responsibility and accountability for the successful management and clinical governance of the ACOs [33]. The Minister of Health will appoint the Chairman of each ACO Board. According to AlMubarak et al. (2021), for instance, the Eastern Province's E1 cluster, which consists of medical centres and PHCs, is run by a consultative organisation that primarily draws its resources from King Fahd Specialist Hospital. This strategy represents a dramatic turn in the direction of commercialisation in Saudi Arabia's healthcare system, given the larger objectives of Vision 2030 for both economic and social development [14]. Likewise, this transformation has been designed based on the principles to empower individuals and their families with the ability to manage their health, enable individuals to be well-informed and in control of their health by providing them with knowledge as part of their treatment, and completely incorporate the health system from the perspective of the populace. Maintaining the health of the entire population through a preventive approach, as opposed to a solely curative approach to health provision, and delivering treatment in a manner that is both patient-friendly and outcome-oriented, while ensuring that patients are not over- or under-treated [25].

Policies Promoting PHCs

Although their effects have not been thoroughly assessed, Saudi Arabia has implemented several national programs and policies to improve PHC services. These programs include specialised clinics, strengthening family medicine approaches, screening/early detection programs, tobacco control programmes, human resources for health recruitment programmes, primary mental health, scaling of family physicians’ postgraduate training, family physician refresher training, and customer service programmes [42].

The study by Alfaqeeh et al. (2017) revealed important variables affecting this disparity, including accessibility to health preventative programs, sanitation, and distance. Improving amenities implemented in rural regions may result in better health conditions and a more equal medical system throughout the region [24]. Research by Alnasser et al. (2023) found that the Saudi Arabian medical sector has worked hard to alleviate the problems caused by non-urgent ED consultations [23]. The nation has constantly classified patients according to the urgency of their ailments for more than 16 years since the majority of institutions adopted the Canadian Triage and Acuity Scale (CTAS). The healthcare system has attempted to improve the standard of care for individuals in dire circumstances by distinguishing between urgent and non-urgent conditions. People still often attend EDs because they believe their ailments are more serious than they are, even if there are complete PHCs that are open to the public. The expense of non-urgent ED appointments in the nation could be lessened by initiatives that emphasise the importance of PHCCs and remove access obstacles [23].

Al-Rebdi et al. (2021) observed that Saudi Arabia has, since 1989, worked to safeguard and maintain the dignity of patients by establishing a Patients' Bill of Rights (PBR) under the Ministry of National Guard Health Affairs (MNGHA), in compliance with worldwide medical regulations [22]. The obligations that healthcare institutions have to patients and their loved ones are described in this policy. Despite these initiatives, national polls indicate that there are still significant differences in the understanding and utilisation of these rights.

Saudi Arabia has implemented stringent national policies in compliance with the Global NCD Action Plan principles as a reaction to the rising risk of NCDs [16]. These programs have been incorporated by the nation's Ministry of Health into both its policies and the larger Saudi Vision 2030. They have highlighted the significance of PHCs in tackling NCD protection and regulation throughout the National Disease Control Programs (NDCPs).

However, there are several grievances regarding the numerous shortcomings of the private sector, including inadequate infrastructure without registration [33], subpar equipment, a lack of qualified staff [43], subpar service conditions [33], higher treatment costs, incorrect disease diagnosis, commission-based services, overservicing, excessive prescription of drugs and tests, and overuse. These grievances have led to a lack of trust and confidence in the private healthcare sector among the general population. Additionally, these issues highlight the need for stricter regulations and oversight to ensure quality and affordable healthcare services for all individuals. 

Even in a global setting, the evidence regarding the relative efficacy, efficiency, and overall benefits of privatising the healthcare industry has been largely inconclusive, with no study presenting conclusive evidence in favour of or against greater private-sector participation [44]. As a result, it is impossible to quantify the value of the private sector's contribution to the delivery of healthcare in a consistent way [37]. The lack of conclusive evidence regarding the benefits of privatising the healthcare industry highlights the complexity of evaluating its impact. Different countries and contexts may yield varying results, making it challenging to draw definitive conclusions. Therefore, further research and analysis are necessary to understand better the role and value of private sector involvement in healthcare delivery.

## Conclusions

Saudi Arabia has advanced its PHC facilities significantly. This ensures more effective provision of healthcare by increasing affordability and considerably reducing the strain on secondary and tertiary emergency departments at hospitals. Cutting-edge tools for scheduling appointments demonstrates a dedication to expediting immunisations and preventative care, underscoring the significance of preemptive healthcare administration. Furthermore, these centres' thoughtful geographic placement shows a deliberate attempt to close disparities between urban and rural locations, guaranteeing fair accessibility for all demographic groups, as Saudi Arabia's notable advancement towards a comprehensive healthcare structure is its devotion to Universal Health Coverage. Nevertheless, ongoing consideration and all-encompassing approaches are required to address enduring issues with patient fulfillment, care coordination, excellent service, and long-term disease control. Encouraging various patient perspectives and matching treatment situations with high contentment ratings are critical to the achievement of these revolutionary healthcare innovations.
